# An Optical Water Type-Based Deep Learning Framework for Enhanced Turbidity Estimation in Inland Waters from Sentinel-2 Imagery

**DOI:** 10.3390/s25206483

**Published:** 2025-10-20

**Authors:** Yue Ma, Qiuyue Chen, Kaishan Song, Qian Yang, Qiang Zheng, Yongchao Ma

**Affiliations:** 1 School of Geomatics and Prospecting Engineering, Jilin Jianzhu University, Changchun 130118, China; chenqiuyue@student.jlju.edu.cn (Q.C.); zhengqiang0222@student.jlju.edu.cn (Q.Z.); mayongchao@student.jlju.edu.cn (Y.M.); 2 State Key Laboratory of Black Soils Conservation and Utilization, Northeast Institute of Geography and Agroecology, Chinese Academy of Sciences, Changchun 130102, China; songkaishan@iga.ac.cn (K.S.); yangqian@iga.ac.cn (Q.Y.)

**Keywords:** turbidity estimation, optical water types, deep learning model, Sentinel-2 imagery

## Abstract

**Highlights:**

**What are the main findings?**

- A novel framework that integrates the fuzzy c-means method with a weighted blending CNN-RF deep learning model for accurate turbidity estimation based on optical water types was effectively implemented and validated using Sentinel-2 data.
- The OWT-based deep learning model achieves robust and generalizable turbidity predictions with high accuracy and effectively retrieves turbidity to capture the continuous spatial distribution characteristics of inland waters.

**What is the implication of the main finding?**

- This study provides a practical and accurate method for facilitating the application of deep learning models based on the optical classification of inland waters in turbidity estimation.
- The validated framework and methods enhance the operational capabilities of remote sensing for water quality monitoring and provide algorithmic support for a more comprehensive understanding of aquatic environmental conditions and ecosystem dynamics.

**Abstract:**

Turbidity is a crucial and reliable indicator that is extensively utilized in water quality monitoring through remote sensing technology. The development of accurate and applicable models for turbidity estimation is essential. While many existing studies rely on uniform models based on statistical regression or traditional machine learning techniques, the application of deep learning models for turbidity estimation remains limited. This study proposed deep learning models for turbidity estimation based on optical classification of inland waters using Sentinel-2 data. Specifically, the fuzzy c-means (FCM) clustering method was employed to classify optical water types (OWTs) based on their spectral reflectance characteristics. A weighted sum of the turbidity prediction results was generated by the OWT-based convolutional neural network-random forest (CNN-RF) model, with weights derived from the FCM membership degrees. Turbidity for four typical waters was mapped by the proposed method using Sentinel-2 images. The FCM method efficiently classified waters into three OWTs. The OWT-based weighted CNN-RF model demonstrated strong robustness and generalization performance, achieving a high prediction accuracy (*R*^2^ = 0.900, *RMSE* = 11.698 NTU). The turbidity maps preserved the spatial continuity of the turbidity distribution and accurately reflected water quality conditions. These findings facilitate the application of deep learning models based on optical classification in turbidity estimation and enhance the capabilities of remote sensing for water quality monitoring.

## 1. Introduction

Lakes and reservoirs, as important inland water resources, significantly influence ecological balance, socioeconomic development, and human well-being [1,2,3]. They absorb runoff, regulate regional climates, and provide freshwater resources for drinking, agricultural irrigation, and industrial production [4]. Additionally, these water bodies are essential for hydropower generation, flood control, aquaculture, and recreational activities [5]. Lakes and reservoirs are critical for maintaining ecological and socioeconomic sustainability [6]. However, due to increasing anthropogenic activities and the impacts of climate change, these water bodies have experienced issues such as area reduction and water quality deterioration, which threaten aquatic ecosystems [7]. Regular water quality monitoring is essential for understanding environmental changes and supporting the formulation of effective strategies for aquatic ecosystem protection. Turbidity, which measures the degree to which suspended particles in water scatter light, has become a widely used indicator of water quality conditions. It offers a simple, economical, and practical alternative to direct measurements of suspended sediment [8,9,10]. Turbidity is closely related to optically active substances in water, such as phytoplankton, non-algal particles, and colored dissolved organic matter [11,12,13]. Elevated levels of suspended particulate matter or chlorophyll-a concentrations can restrict light penetration, increase turbidity, and signal a decline in water quality. Thus, effectively and accurately quantifying water turbidity is a necessary and fundamental task for enhancing the understanding of water quality in lakes and reservoirs.

Remote sensing technology has been regarded as an advanced tool for monitoring water turbidity since 1980, when Moore evaluated the feasibility of satellite data [14,15]. Numerous satellites have been launched to provide extensive remote sensing data, which has been successfully applied to mapping water turbidity in inland waters [2,16,17]. For instance, various satellite remote sensing instruments, such as Geostationary Ocean Color Imager (GOCI), Moderate Resolution Imaging Spectroradiometer (MODIS), Ocean and Land Colour Instrument (OLCI), and Medium Resolution Imaging Spectrometer (MERIS), are capable of acquiring optical measurements related to turbidity and generating images with medium spatial resolution exceeding 100 m [6,18,19,20,21]. However, these datasets exhibit limitations in good spatial resolution for monitoring smaller water bodies and sufficient spectral response for estimating water turbidity. The Landsat series of satellites, deployed relatively early, has provided data with improved spatial and spectral resolution, enabling long-term monitoring of water turbidity since the 1970s. Remote sensing data from the MSI (MultiSpectral Instrument) sensor aboard Sentinel-2 satellites offer higher spatial resolution (up to 10 m) and more sensitive spectral bands, making them more suitable than other sensors for estimating water turbidity across a wide range of water bodies, including smaller ones, within the visible and near-infrared spectral ranges. Additionally, Sentinel-2 data provide high temporal resolution (acquired every 2–5 days via Sentinel-2A and 2B), thereby facilitating more accurate temporal alignment with in situ measurements [14]. Moreover, non-satellite remote sensing data obtained from near-surface sensing platforms have also been used for turbidity estimation. Unmanned aerial vehicles (UAVs) equipped with a multi-spectral camera or a hyperspectral imager are convenient and effective to provide more detailed image data for turbidity monitoring [22]. The ground spectral data measured by portable spectrometers (e.g., Analytical Spectral Devices (ASD) spectrometer) can be flexibly used on board to detect the water surface reflectance with the highest spectral resolution [23]. However, data obtained from aircraft or ground measurements involve a higher cost and suffer from limited capacity to comprehensively observe continuous spatiotemporal distribution maps of water turbidity across large areas [24]. Consequently, generating more accurate water turbidity products from Sentinel reflectance products is more suitable and holds significant potential for operational and regular water quality monitoring applications.

Developing accurate, robust, and practical models for turbidity estimation is a foundational task in remote sensing technology. Empirical models based on statistical regression algorithms are commonly developed to establish relationships between water turbidity and sensitive spectral reflectance derived from various satellite remote sensing data [25]. Although empirical models are relatively simple to develop, they often lack robustness and generalization capability. In addition, semi-analytical models, which are based on the inherent optical properties of water, require more complex computational procedures and are more challenging to implement. Compared to these traditional methods, data-driven machine learning methods have become more promising methods for remotely estimating water turbidity. Machine learning methods offer a significant advantage in data mining and exhibit superior performance in modeling the nonlinear relationships between turbidity and spectral reflectance [19,26]. Several machine learning methods, including random forest regressor (RFR), extreme gradient boosting (XGBoost), support vector regression (SVR), and back-propagation neural network (BP), have been successfully applied for turbidity prediction in inland waters. The rapid advancement of deep learning algorithms has further enabled the development of advanced models for water quality assessment [27,28,29,30,31]. Compared to machine learning models, deep learning algorithms excel in constructing deep network architectures with multiple hidden layers. These methods progressively extract complex data relationships through iterative training, thereby significantly enhancing prediction accuracy [32]. Several researchers have applied deep learning algorithms, such as a convolutional neural network (CNN) and a deep neural network (DNN), to accurately estimate water quality indicators for aquatic ecological environment assessment [33,34,35].

However, challenges persist in constructing deep learning models for water turbidity estimation, as predictive performance is often affected by uncertainties arising from limitations in model generalization and dataset quality. In particular, lakes and reservoirs, categorized as optically complex Case II waters, pose significant challenges to developing a universal learning model based on samples from diverse water bodies. This is primarily due to the complex interplay of optical signals from chlorophyll, suspended sediments (SPM), and colored dissolved organic matter (CDOM) in these waters [36,37,38,39]. To overcome the heterogeneity of water optics, several studies have proved that waters can be classified into distinct optical water types (OWTs) based on observed spectra before model construction [40]. Since model performance is closely related to the optical properties of waters, classifying waters and developing specific estimation models for each OWT can enhance model accuracy and yield more reliable estimation results [14,41]. In general, optical water classification methods mainly include clustering methods based on the magnitude and shape of remote sensing reflectance, and bio-optical threshold methods based on the inherent optical properties (IOPs) of water (i.e., absorption and scattering) [11,42]. Among them, clustering methods are widely employed for water classification through automated unsupervised classification techniques (e.g., hierarchical clustering, k-means clustering, and fuzzy c-means clustering) [43,44,45,46], and empirical threshold partitioning methods for specific spectra [47].

Although these optical water classification methods have demonstrated efficiency in estimating chlorophyll or TSM, there remains a lack of deep knowledge regarding their potential applicability in capturing the Sentinel-2 characteristic spectrum of different water bodies for turbidity estimation. Moreover, most studies rely on uniform estimation models developed using statistical regression algorithms or traditional machine learning techniques, with limited exploration of deep learning approaches for water turbidity estimation. Consequently, to improve turbidity estimation for water quality monitoring in inland waters, this study proposes deep learning models for turbidity estimation based on specific water optical types using Sentinel-2 data. To achieve this goal, the specific research objectives are as follows: (1) classify water types for turbidity estimation based on the spectral reflectance characteristics of Sentinel-2 data; (2) establish deep learning methods for estimating water turbidity based on specific water optical types; (3) apply the established models to invert water turbidity from Sentinel-2 imagery across several representative lakes and reservoirs.

The rest of the paper is structured as follows: Section 2 outlines the geographical conditions of the study area, the data resources and processing procedures, and the basic theory of the algorithms used within this study. Specifically, the key algorithms include the optical water classification method, the proposed turbidity estimation method, and the evaluation criteria of model performance. The results are presented and discussed in Section 3 and Section 4. Finally, Section 5 summarizes the main conclusions.

## 2. Materials and Methods

### 2.1. Study Area

**Figure 1:**
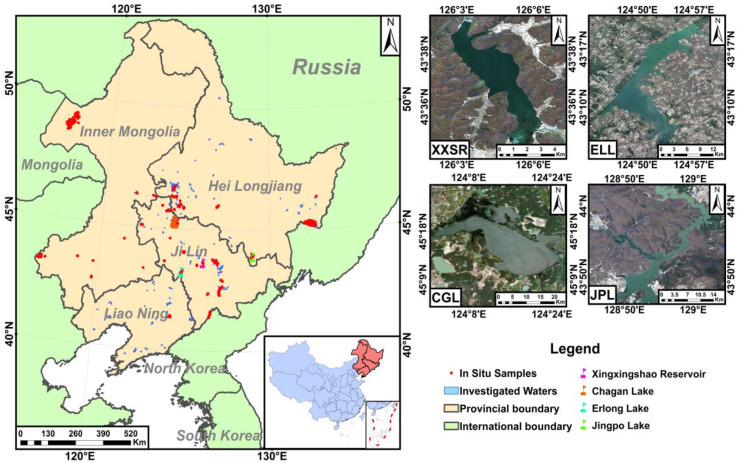
Locations of four typical lakes and reservoirs and the spatial distribution of sampling points in waters where in situ measurements were made in Northeast China.

The study area is situated in Northeast China (Longitude: 118°53′ E–135°05′ E, Latitude: 38°43′ N–53°33′ N), encompassing Liaoning Province, Jilin Province, Heilongjiang Province, and the eastern part of Inner Mongolia Autonomous Region, with a total area of 1,240,897 km^2^ (Figure 1). Surrounded by the Greater Khingan, Lesser Khingan, and Changbai Mountains, the region features a relatively elevated topography. Major river systems, including the Liaohe River, Songhua River, and Nenjiang River, form the low-lying Northeast China Plain, including the Songnen Plain, Sanjiang Plain, and Liaohe Plain. The region contains hundreds of rivers, lakes, and reservoirs, which provide vital support for people’s livelihoods as well as industrial and agricultural production. Moreover, this region is characterized by a temperate monsoon climate with distinct four seasons. The annual average temperature ranges from 2 °C to 6 °C, and the annual precipitation is 350–700 mm. Winters in Northeast China are long and severe, with a freezing period of up to 5–6 months. The prolonged freezing period significantly reduces the capacity of water bodies to degrade pollutants and undergo natural self-purification, resulting in a marked deterioration of water quality [48]. As a crucial national industrial and grain production base, the region has several shallow waters that are mesotrophic or eutrophic due to the influence of intensive human activities. To conduct a comprehensive evaluation of model performance, four representative and important lakes and reservoirs, namely, Xingxingshao Reservoir (XXSR), Chagan Lake (CGL), Erlong Lake (ELL), and Jingpo Lake (JPL), were selected to retrieve water turbidity from Sentinel-2 image data for detailed analysis.

### 2.2. Data Acquisition and Processing

#### 2.2.1. Field Survey and Laboratory Measurements

Field sampling was conducted annually from April to October between 2018 and 2024 to monitor water turbidity in selected lakes and reservoirs across Northeast China. During each sampling campaign, the geographic coordinates of the sampling sites were accurately recorded using a Trimble PXRS GPS receiver (Trimble Navigation, Inc., Sunnyvale, CA, USA). Water samples were collected at a depth of 0.5 m below the surface, stored in a portable refrigerator at 4 °C, and transported to the laboratory for subsequent analysis within 7 days. In the laboratory at a controlled temperature of 20 ± 2 °C, artificial turbid water of 400 NTU was prepared. Distilled water was filtered through a 0.2 μm glass fiber membrane to obtain water free of turbidity. Standard turbidity solutions were prepared by diluting the turbid water using the filtered distilled water. The turbidity of water samples was measured using a UV–VIS spectrophotometer (SHIMADZU UV-2600, SHIMADZU, Kyoto, Japan) at a wavelength of 680 nm with a 3 cm quartz cuvette. The measured turbidity of samples was determined using the calibration curve derived from the absorbance of standard solutions at 680 nm.

#### 2.2.2. Sentinel-2 MSI Data Acquisition and Processing

The Sentinel-2 mission, part of the Copernicus Programme, comprises two identical satellites operating in tandem to support the monitoring of changes on the Earth’s surface. Sentinel-2 is designed to provide a large swath width of 290 km and a high revisit frequency of 2–3 days at mid-latitudes, enabling frequent monitoring of surface water conditions [49]. The optical Multi-Spectral Instrument (MSI) onboard Sentinel-2 measures the reflected radiance in 13 spectral bands, ranging from the VNIR (Visible and Near-Infra-Red) to the SWIR (Short Wave Infra-Red). The MSI sensor features three distinct spatial resolutions, each corresponding to specific spectral bands. Specially, there are four classical VNIR bands with a 10 m spatial resolution: Blue (493 nm), Green (560 nm), Red (665 nm), and NIR (833 nm); four narrow bands in the VNIR vegetation red edge spectral domain (704 nm, 740 nm, 783 nm and 865 nm) and 2 wider SWIR bands (1610 nm and 2190 nm) with a 20 m spatial resolution; three bands primarily focused on cloud screening and atmospheric correction (443 nm for aerosols and 945 nm for water vapour) and cirrus detection (1374 nm) with a 60 m spatial resolution.

In this study, Sentinel surface reflectance images were obtained through the Google Earth Engine (GEE) platform. Cloud-free Sentinel-2 images that corresponded to the in situ data within a ±7-day temporal window were selected [14]. The reflectance of the central pixel was extracted by using the average surface reflectance within a 3 × 3 window size, and this central pixel was matched with the in situ turbidity measurement points. A total of 668 in situ sampling points were successfully matched with Sentinel-2 imagery over the period from 2018 to 2024. These data were used for optical water classification and model construction. The statistical information on water turbidity of the sampling points across different sampling years (2018–2024) is shown in Table 1.

The point data was primarily utilized for optical water classification. These data were also used to construct machine learning models, which served as a control group for evaluating the performance of deep learning models. In addition, with the Sentinel-2 image pixel corresponding to the field observation point set as the central pixel, image patches are segmented by defining window sizes as specific side lengths, which were generated for constructing deep learning models. To retrieve water turbidity information for selected lakes and reservoirs, water bodies were extracted using manual visual interpretation of Sentinel-2 images acquired in 2024. The water boundaries were created by vectoring and could be effectively and directly converted into a water body map.

### 2.3. Methodology

#### 2.3.1. Fuzzy C-Means Algorithms for Optical Water Classification

The fuzzy c-means (FCM) algorithm, a soft clustering method, was selected for optical water classification. This approach generates multiple clusters, allowing each data point to belong to more than one cluster with varying degrees of membership. Membership grades are introduced to quantify the extent to which each data point belongs to a particular cluster [50]. Consequently, data points with lower membership grades exhibit a weaker association with a given cluster compared to those with higher grades. This method aims to minimize the distance between the data points and their corresponding cluster centroids [51]. The objective function, as presented in Equation (1), is continuously minimized through iterative updates of the membership grades and cluster centroids. The computation of the cluster centers is the weighted average of the membership values, as defined in Equation (2) [46]. The iterative process terminates when either the maximum number of iterations is reached or the residual change falls below a predefined minimum threshold. Each data point can ultimately be assigned to one cluster based on the maximum membership grade. The FCM algorithm returns a set of fuzzy clusters and a membership matrix containing the membership grades of each data point for every cluster [52].

(1) $$J_{FCM}=J(U,v)=\sum_{i=1}^{N}\sum_{j=1}^{C}u_{ij}^{m}d_{ij}^{2}$$

(2) $$u_{ij}={\left(\sum_{k=1}^{C}{\left(d_{ij}/d_{ik}\right)}^{2/m-1}\right)}^{-1}$$

where *N* is the number of samples; *C* denotes the number of clusters; *u_ij_* is an *i* × *j* matrix with membership degrees; *d* is the Euclidean distance; and *m* = 2 is fuzzification degree.

Cluster validity measures are essential for assessing clustering outcomes and determining the optimal number of clusters. In this study, the partition entropy (PE) index and Xie-Beni (XB) index were jointly utilized to determine the number of clusters when both indices reached their minimum values [53,54]. The corresponding formulas are presented in Equations (3) and (4). These two measures considered the fuzziness of membership grades and the compactness and separateness of clusters.

(3) $$V_{PE}=-\frac{1}{N}\sum_{i=1}^{N}\sum_{j=1}^{K}u_{ij}\log_{a}(u_{ij})$$

(4) $$V_{XB}=\frac{\sum_{i=1}^{N}\sum_{j=1}^{K}u_{ij}^{m}d_{ij}^{2}}{N\min_{i,j}\left(d_{ij}^{2}\right)}$$

where *K* denotes the number of clusters to be evaluated; other parameters are as defined in the aforementioned formulas.

#### 2.3.2. Base Regressor Algorithms for Turbidity Estimation

A convolutional neural network (CNN) is a widely used deep learning model that employs forward and backward propagation algorithms to construct a deep neural network. The fundamental components of a CNN are multiple convolutional layers. Several two-dimensional image patches with a specific window size were fed into the first layer, referred to as the input layer. The CNN window size determines the size of image patches used to train the model, thereby affecting its feature extraction capability, computational efficiency, and final performance. A convolutional layer performs the dot product between the input two-dimensional vector and the convolution kernel that contains learnable weights and biases. Subsequently, an activation function is applied to introduce a nonlinear transformation. In the convolutional layers, various feature maps are generated as several different filters slide across the input vector. Following the convolutional layers, the pooling layer serves to reduce computational complexity and the number of parameters, thereby mitigating the risk of overfitting. Specifically, the operations performed in the convolutional layers can be expressed by Equation (5) [55,56].

(5) $$O^{l}=pool_{p}(\sigma (O^{l-1}*W^{l}+d^{l}))$$

where *O^l−^*^1^ denotes the input feature map to the *l*th layer; *W^l^* and *d^l^* represent the weights and biases of the layer, respectively, which convolve the input feature map through linear convolution; and *σ*(·) indicates the non-linearity function outside the convolutional.

Fully connected layers, as the final layer of the CNN, receive inputs corresponding to the flattened one-dimensional vector derived from the last pooling layer. Complex CNN models often face the challenge of overfitting. Several regularization techniques, such as dropout (i.e., randomly deactivating some neurons) and batch normalization (i.e., normalizing the input vector), are commonly employed to mitigate overfitting [57]. Within a CNN architecture, these layers are systematically integrated, with the number of each layer type typically determined by the specific requirements of the application. During forward propagation, the CNN model extracts abundant data information by transforming the input data through successive convolutional blocks, extracting increasingly abstract and informative features at each stage, and ultimately producing the final output through the fully connected layers. Throughout this process, the model’s weights and biases are initially set to random values. In the backward propagation process, the CNN model iteratively updates the learnable parameters by minimizing the loss function, which quantifies the error margin between the model’s prediction and the actual target value [58]. Through this iterative optimization process, the CNN gradually enhances its predictive accuracy.

In addition, applying the CNN model to solve regression problems requires the selection of an appropriate activation function. The widely used linear activation function enables the neural network to map inputs directly to outputs without undergoing complex mathematical transformations. Although it demonstrates efficiency in simpler regression tasks, it is limited in its ability to capture complex nonlinear relationships. In this study, the recognized promising regression method, the random forest (RF) regressor, is integrated with the CNN model to enhance the predictive capability for water turbidity.

Random forest is a tree-based ensemble machine learning method [59]. This method combines several individual classification and regression trees (CARTs) through bagging ensemble technology. Each CART, constructed with bootstrap samples rather than the entire original dataset, recursively partitions the feature space at each node to group similar targets and ultimately form binary trees [60]. In regression tasks, the loss function commonly uses mean squared error, as defined in Equation (6), as a criterion to minimize for splitting each node. Finally, the random forest regressor generates the prediction result by averaging the regression prediction results of all individual trees.

(6) $$H(Q_{m})=\frac{1}{N_{m}}\sum_{y\in Q_{m}}(y-{\bar{y}}_{m}{)}^{2}$$

where *H*(·) is the loss function that mean square error, *Q_m_* represents the datasets at node *m*, *N_m_* is the number of samples at node *m*, and *y* and ${\bar{y}}_{m}$ respectively represent the original and mean observed turbidity values at node *m*.

To compare the accuracy of the proposed model with that of other models, we selected several other popular, high-performance machine learning models in this study, including support vector regression (SVR), back-propagation neural network (BP), and extreme gradient boosting (XGBoost). Support Vector Regression (SVR) is a popular supervised learning method derived from statistical learning theory, and it can be applied to regression tasks. SVR is able to transform training datasets into a higher-dimensional feature space through the use of a kernel function [61]. BP is a flexible algorithm for modeling based on a multi-layer perceptron, which consists of input, hidden, and output layers, each comprising a set of neurons. BP utilizes a back-propagation algorithm to dynamically update the learnable parameters based on back-propagation errors [62]. XGBoost is a boosting-based ensemble learning method that consists of several regression decision trees as base regressors. It constructs a new decision tree based on the negative gradient direction of the loss function from the previous decision tree, and gradually reduces the loss through iterative methods. The average value of the predicted values from all base regressors is adopted as the final prediction result [63].

#### 2.3.3. Research Framework for Turbidity Estimation Based on the Proposed Models

**Figure 2:**
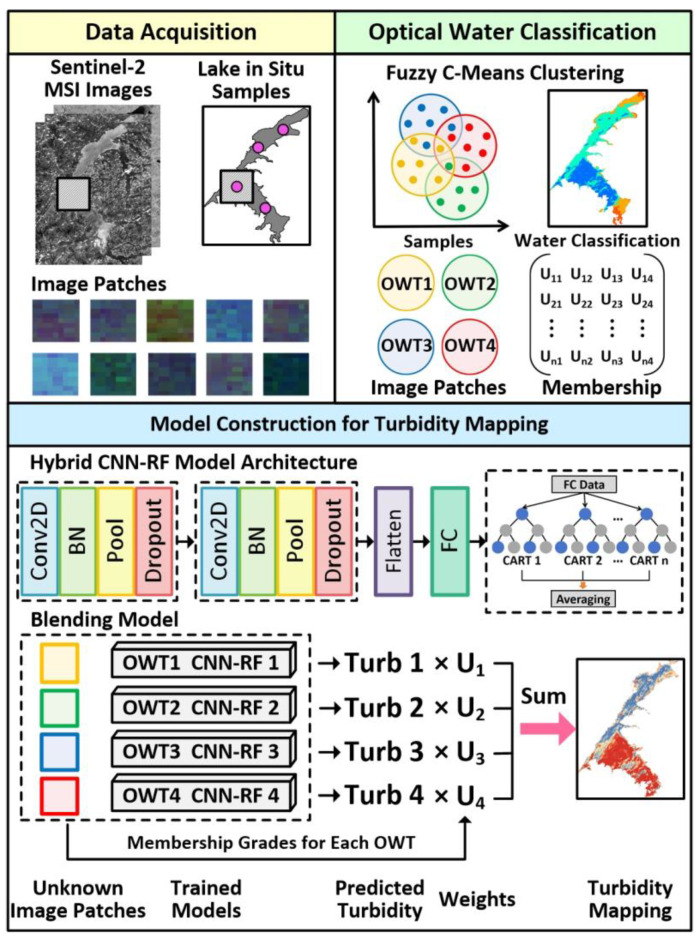
Flowchart of turbidity estimation based on optical water type classification using Sentinel-2 MSI data. Conv2D, BN, Pool, Dropout, Flatten, and FC represent the convolutional layer, batch normalization layer, max-pooling layer, dropout layer, flatten layer, and fully connected layer, respectively.

As illustrated in Figure 2, the primary workflow for turbidity estimation in this study comprised three key components: data acquisition, optical water classification, and deep learning model construction with spatial turbidity mapping. In this paper, the models were constructed using the Python 3.10 programming language within the PyCharm 2022.2.1 (JetBrains N.V., Amsterdam, The Netherlands) software. The environment configuration was based on Anaconda 3, where TensorFlow 2.15 was installed as the deep learning framework. Each process was implemented as follows:

(1) Two types of datasets were generated by matching Sentinel-2 images with in situ points, comprising the point data with spectral reflectance and the two-dimensional image patches for constructing deep learning models.
(2) The fuzzy c-means cluster method was applied to the point data with Sentinel-2 spectral reflectance for optical water classification. The FCM method automatically partitioned samples based on the similarity of spectral characteristics. After identifying the clusters, the spectral reflectance of cluster centroids and a matrix encompassing each data point’s membership grades were calculated for each cluster. The dominant OWT for each point was determined as the cluster with the highest membership value. Subsequently, two-dimensional image patches corresponding to the classified sample points were grouped according to their assigned OWTs and utilized to train distinct turbidity prediction models.
(3) We combined the CNN model and RF algorithm to form the CNN-RF model, which was employed to predict the water turbidity based on OWTs. To balance model complexity and generalization, the CNN framework incorporated two identical convolutional blocks. Each block consisted of a convolutional layer, a batch normalization layer, a max-pooling layer, and a dropout layer. The convolutional layers in the first and second blocks used 32 and 64 filters, respectively, with a 3 × 3 window size to generate feature maps. These two blocks were followed by a flattening layer and a fully connected layer containing 128 neurons. During the convolutional process, the spatial and spectral features of the input images were extracted. Subsequently, the output of the fully connected layer was supplied as input to a random forest algorithm, which served as a regressor for predicting water turbidity.
(4) The FCM algorithm was applied to classify the samples to be predicted into a specified number of OWTs, with each type assigned a corresponding membership value. Water turbidity was predicted using pre-trained CNN-RF models for each OWT, yielding turbidity estimates corresponding to each OWT. The Euclidean distance was employed to assess the similarity between the sample’s reflectance and the spectral centroid of each OWT. A smaller distance indicates a higher similarity, which leads to a higher membership value for that OWT. These membership values were subsequently used as weighting factors for the turbidity predictions generated by the respective CNN-RF models. The weighted sum of all OWT-based turbidity predictions generated the final blending result.
(5) For the application of Sentinel-2 imagery in water type classification and turbidity mapping, the image spectra were used as input for the FCM and CNN-RF models. For water classification, each pixel was assigned to a specific water type based on the maximum membership grade, which was derived from membership functions that calculated the distance between the reflectance of individual pixels and the spectral centroids of the in situ measured data. For turbidity mapping, each classified pixel with membership grades was predicted by the OWT-based CNN-RF models, and the blending retrieval result was the weighted sum of all OWT-based turbidity predictions using membership values.

#### 2.3.4. Model Accuracy Evaluation

The predictions by different models were compared using the coefficient of determination (*R*^2^) and root mean square error (*RMSE*) as evaluation metrics. *R*^2^ represents the proportion of total dependent variable variance explained by the independent variables in the model, thus assessing the effectiveness of a regression model in predicting the outcome variable. *RMSE* measures the difference between the predicted and actual values. A smaller *RMSE* suggests a smaller discrepancy between the predicted and actual values, indicating more accurate predictions by the model. Their specific calculation formulas are as follows:

(7) $$R^{2}=1-\sum_{i=1}^{n}{\left(y_{i}-y_{i}\prime \right)}^{2}/\sum_{i=1}^{n}{\left(y_{i}-{\bar{y}}_{i}\right)}^{2}$$

(8) $$RMSE=\sqrt{\sum_{i=1}^{n}{\left(y_{i}-y_{i}\prime \right)}^{2}/n}$$

where *n* represents the number of samples, while $y_{i}$ and $y_{i}\prime$ refer to the observed and predicted turbidity values, respectively. $\bar{y}$ is the average of the observed turbidity values.

## 3. Results

### 3.1. Spectral Fuzzy Clustering Using in Situ Data

**Figure 3:**
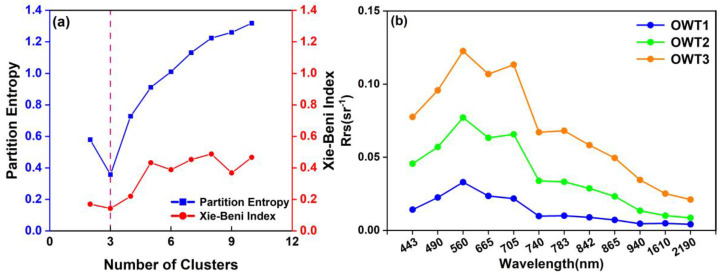
Assessment of cluster numbers based on cluster validity measures and the spectral reflectance of cluster centroids corresponding to different OWTs. (**a**) illustrates the values of PE and XB indices calculated based on different numbers of clusters; (**b**) illustrates the spectral reflectance of cluster centroids for distinct OWTs.

Figure 3a shows the PE and XB indices based on different numbers of clusters. As the number of clusters increased, the PE and XB indices showed monotonically increasing trends. At higher values, these two cluster validity measures indicate a higher degree of fuzziness and greater overlap in the clusters. Thus, the optimal number of clusters was determined as 3 since it exhibited the minimum PE and XB. With three clusters specified, the relationship between data points and cluster centers was overall optimal in terms of the fuzziness of membership grades and the compactness and separateness of clusters.

The mean spectral reflectance of cluster centroids reflected the apparent optical properties (AOPs) of different OWTs, which depended on the absorption and scattering properties of the water constituents. As shown in Figure 3b, these spectral curves of in situ points exhibit the typical optical properties of Case II inland waters. OWT 1 exhibited lower mean spectral reflectance, and its spectral curve was flatter from 665 nm onward compared to that of other OWTs. The turbidity, chlorophyll a (Chl-a), and total suspended matter (TSM) of OWT 1 averaged the lowest at 27.975 NTU, 8.807 μg/L, and 25.271 mg/L, respectively (Table 2). Low particle concentration and water absorption contributed to the spectral reflectance features of OWT1. OWT 2 and OWT 3 produced similar spectral curves, with two reflection peaks around 560 nm and 705 nm and an absorption valley near 665 nm. Specifically, the reflection peaks were mainly caused by the strong scattering of particles from phytoplankton and sediment sources, and the valley around 665 nm can be attributed to the absorption of Chl-a. In addition, OWT 3 exhibited a slightly deeper absorption valley around 665 nm and higher peaks near 705 nm and 783 nm than OWT 2. These differences might stem from the higher Chl-a concentration in OWT 3. However, the spectral curve differences were insignificant, and their mean Chl-a concentrations were relatively close (12.360 μg/L for OWT 2 and 15.590 μg/L for OWT 3; Table 2). Moreover, the mean spectral reflectance of OWT 3 was higher than that of other OWTs, with the highest turbidity (57.214 NTU) and TSM (50.607 mg/L; Table 2).

### 3.2. Turbidity Estimation Model Construction and Performance Evaluation

#### 3.2.1. Base Regressor Construction

**Figure 4:**
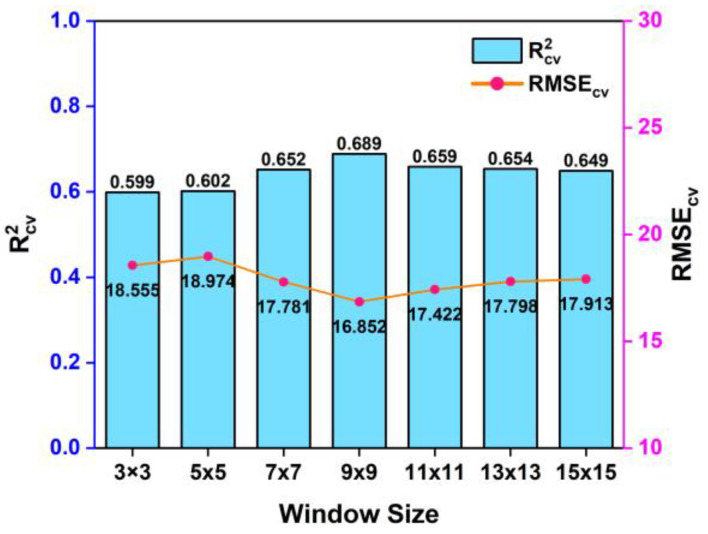
Cross-validation accuracy assessment of CNN models with different window sizes.

The CNN and RF models were combined into the base regressor for the proposed blending algorithm. Figure 4 shows the accuracy of CNN models that utilized input image patches with window sizes of 3 × 3, 5 × 5, 7 × 7, 9 × 9, 11 × 11, 13 × 13, and 15 × 15. Considering the requirement for sufficient samples in the construction of deep learning models, we used the total samples to implement a 3-fold cross-validation method for assessing the influence of window sizes on prediction accuracy. As the window size gradually increased from 3 × 3 to 9 × 9, the cross-validation accuracy *R*^2^ increased from 0.599 to 0.689, while the *RMSE* decreased from 18.555 to 16.852 NTU. The model achieved the best accuracy with a 9 × 9 window size, showing slight accuracy decreases with larger sizes. Thus, the optimal window size was determined as 9 × 9 to acquire training samples by sliding across the images during CNN model construction.

**Figure 5:**
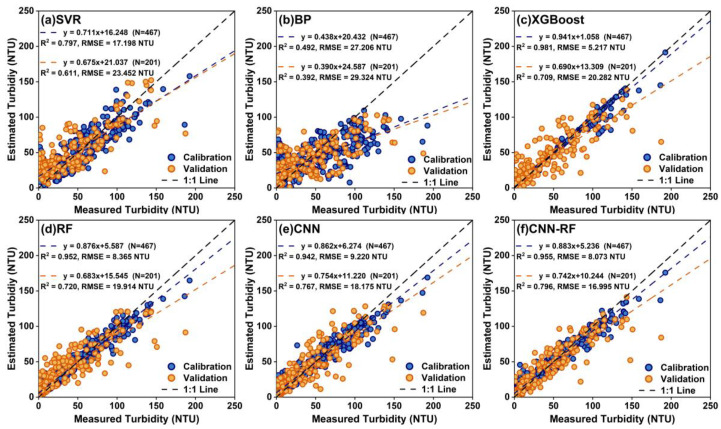
Comparison of accuracy among different base regressor models. (**a**) SVR, (**b**) BP, (**c**) XGBoost, (**d**) RF, (**e**) CNN, (**f**) CNN-RF.

The performance of the base regressor models was evaluated by using 467 samples to construct the model and 201 independent samples to verify the precision. Figure 5 shows the calibration and validation accuracies of the CNN-RF model compared to those of the SVR, BP, XGBoost, RF and CNN models. The tree-based ensemble machine learning models (i.e., XGBoost, RF) and deep learning models (i.e., CNN, CNN-RF) performed well in the calibration set, all with *R*^2^ exceeding 0.94 and *RMSE* below 10 NTU. On the validation set, among these two tree-based ensemble models, the RF model outperformed the XGBoost model, yielding a higher *R*^2^ and lower *RMSE* of 0.720 and 19.914 NTU, respectively. A comparison of the two deep learning models showed that the CNN-RF model outperformed the CNN model, achieving a higher *R*^2^ of 0.796 and a lower *RMSE* of 16.995 NTU. The SVR and BP models exhibited poorer performance in turbidity estimation. However, it should be noted that while deep learning algorithms achieved the highest accuracy, their model operational efficiency was lower than that of machine learning algorithms. According to the scatter distribution, most of the CNN and CNN-RF models’ validation samples clustered around the 1:1 line, with similar slopes (0.754 and 0.742, respectively). However, a minority of sample points remained relatively scattered, indicating a significant prediction deviation.

#### 3.2.2. Performance Evaluation of the Blending Estimation Model Based on OWTs

**Figure 6:**
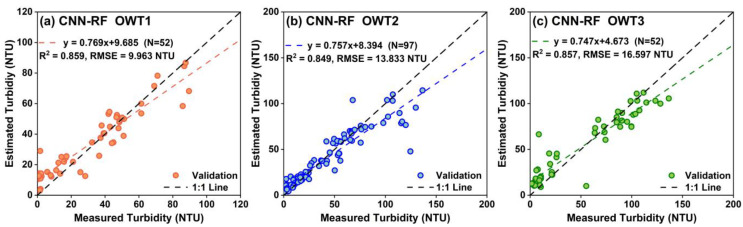
Validation performance of CNN-RF models based on different OWTs. (**a**) CNN-RF model for OWT 1, (**b**) CNN-RF model for OWT 2, (**c**) CNN-RF model for OWT 3.

**Figure 7:**
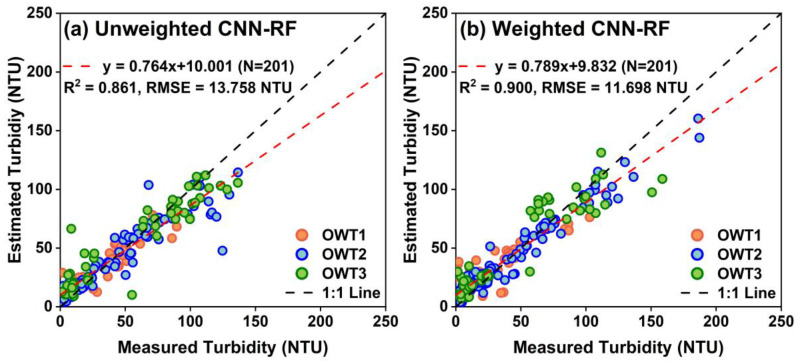
Validation performance of unweighted and weighted CNN-RF models based on OWTs.

The in situ points were divided into three groups based on the FCM method and the spectral reflectance characteristics of the OWTs. All points in each group were divided into calibration and validation sets for modeling and accuracy evaluation. Specifically, the calibration and validation sets included 120 and 52 points in the OWT 1 group, 226 and 97 points in the OWT 2 group, and 121 and 52 points in the OWT 3 group, respectively (Table 3 and Figure 6). The calibration accuracy of the CNN-RF models for each water type is presented in Table 3. The CNN-RF model achieved the highest accuracy for OWT 1 (*R*^2^ = 0.955 and *RMSE* = 5.632 NTU), followed by OWT 3 (*R*^2^ = 0.953 and *RMSE* = 10.180 NTU) and OWT 2 (*R*^2^ = 0.942 and *RMSE* = 8.419 NTU). The CNN-RF model could predict turbidity for different water types, with all calibration accuracies *R*^2^ exceeding 0.94. The turbidity predictions from all OWT-based models were blended using membership weights. As shown in Figure 6, the *R*^2^ and *RMSE* on the validation set are 0.859 and 9.963 NTU for OWT 1, 0.849 and 13.833 NTU for OWT 2, and 0.857 and 16.597 NTU for OWT 3, respectively. The CNN-RF model exhibited significant prediction deviations in the low-value region for OWT 1, with the relative scattered points distributed on both sides of the 1:1 line. All three types of models produced underestimated predictions in the high-value region (turbidity > 85 NTU) but overestimated turbidity in the low-value region (turbidity < 50 NTU). Furthermore, Figure 7 shows that with the CNN-RF model not applying weighted blending to the prediction results of the three OWTs, the overall accuracy indices *R*^2^ and *RMSE* on the validation set are 0.861 and 13.758 NTU, respectively. In contrast, the validation accuracy of the blending CNN-RF model weighted by membership grades improved significantly, with a higher *R*^2^ of 0.900 and a lower *RMSE* of 11.698 NTU. The validation points were more closely aggregated around the 1:1 line, with a relatively high slope of 0.789, and the extent of underestimation or overestimation was mitigated.

### 3.3. Sentinel-2 Image Application

#### 3.3.1. Applicability Analysis of the OWT Classification

**Figure 8:**
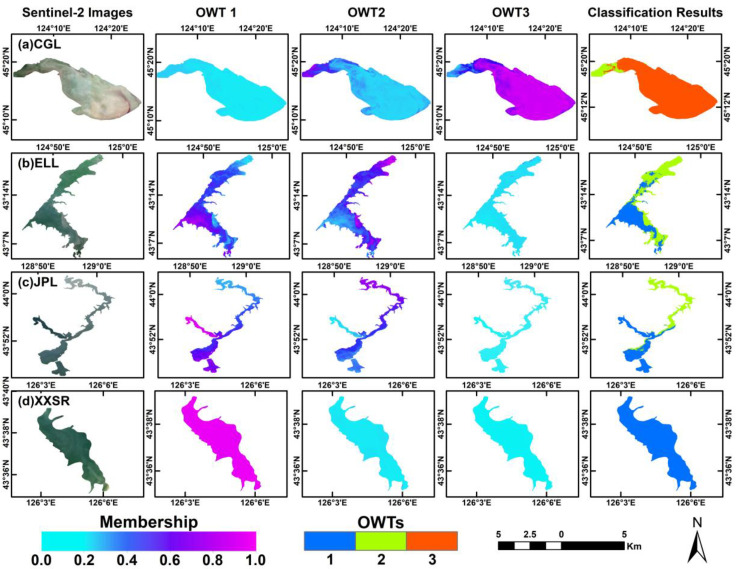
Membership maps and optical water type classification results derived from the FCM model for typical water bodies using Sentinel-2 images. The first column presents Sentinel-2 images, the second to fourth columns show the membership maps of OWT 1, OWT 2, and OWT 3, respectively, and the last column displays OWT classification results. (**a**) Chagan Lake, (**b**) Erlong Lake, (**c**) Jingpo Lake, (**d**) Xingxingshao Reservoir.

The OWTs were mapped from Sentinel-2 images using the membership functions of the FCM method with image spectral as input. Figure 8 presents the membership maps and water type classification maps derived from Sentinel-2 images for the four typical water bodies. The membership maps illustrated the degree to which the pixels belonged to each of the OWTs. According to Figure 8a, the OWT 3 membership map showed strong membership in a spatially continuous region dominating the entire Chagan Lake, while the OWT 2 map showed strong membership in the lake’s northwest corner. The membership for OWT 1 showed weaker affiliation, with values below 0.1. The Chagan Lake water was classified as OWT 2 and OWT 3. Figure 8b,c show that Erlong Lake and Jingpo Lake are both classified into OWT 1 and OWT 2. Specifically, the spatial pattern of water types for these lakes was similar, in which OWT 1 was distributed in the southern part of the water bodies, while OWT 2 was distributed in the northern part. The Xingxingshao Reservoir, as illustrated in Figure 8d, has OWT 1 as the dominant type, which is predominant throughout the entire lake, with the membership values of most pixels exceeding 0.9.

#### 3.3.2. Applicability Analysis of the Turbidity Estimation

**Figure 9:**
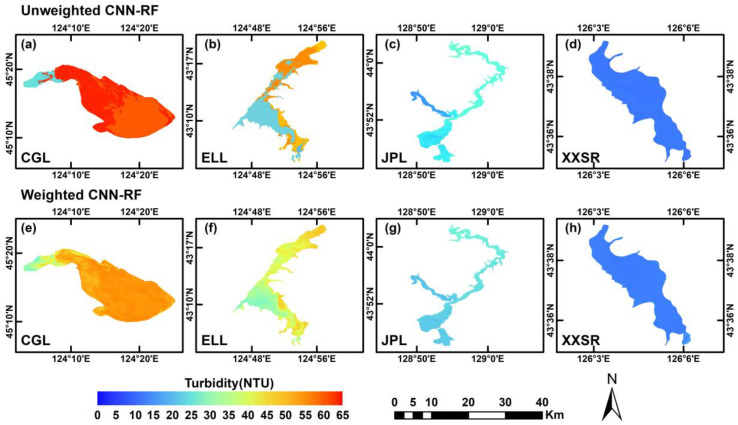
Turbidity spatial distribution maps derived from unweighted and weighted blending models for typical water bodies using Sentinel-2 images. The first and second rows show the turbidity maps generated using unweighted and weighted blending models, respectively. (**a**,**e**) Chagan Lake; (**b**,**f**) Erlong Lake; (**c**,**g**) Jingpo Lake; (**d**,**h**) Xingxingshao Reservoir.

As illustrated in Figure 9, the weighted blending CNN-RF model and unweighted models based on three OWTs are applied to Sentinel-2 images to produce water turbidity maps for four typical water bodies. The spatial distribution patterns of water turbidity generated by these two types of models were similar. Among the four water bodies, Chagan Lake had the highest water turbidity, followed by Erlong Lake, Jingpo Lake, and Xingxingshao Reservoir in that order. Nearly the entire Chagan Lake had relatively higher turbidity with values above 45 NTU, whereas its northwest corner had lower turbidity. In the Erlong Lake and Jingpo Lake, relatively higher turbidity was primarily distributed in their northern parts. The water in Xingxingshao Reservoir exhibited the lowest turbidity, approximately 10 NTU. In addition, the turbidity maps derived from the weighted blending model could effectively avoid discontinuous boundaries, especially in water body regions with significant turbidity variations, such as Chagan Lake (Figure 9e) and Erlong Lake (Figure 9f).

## 4. Discussion

### 4.1. Effectiveness of the FCM Method for OWT Classification

Several studies have demonstrated that a universal algorithm for water bodies with different optical characteristics often fails to deliver accurate predictions. Thus, we classified the water types prior to model construction. With intermixed optical signals from optically active substances in water, assigning unknown samples to definite classes based on spectral characteristics is limited in effectiveness. This study adopted a fuzzy classification approach, the FCM method, to efficiently and automatically assign observations to multiple OWTs with varying membership grades. Two cluster validity indices were used to effectively determine an appropriate number of clusters. The FCM method successfully distinguished spectral differences among waters with distinct optically active substances and turbidity levels. The grouped points provided better datasets for constructing the turbidity estimation models.

Nevertheless, some uncertainties remained in the clustering outcomes. The clustering results might have been affected by insufficient in situ data, as it was not possible to adequately characterize and distinguish all water conditions. In addition, the limited spectral resolution of the Sentinel-2 MSI sensor might omit detailed spectral characteristics that could help identify the absorption and scattering properties of the waters. This unsupervised clustering approach, which utilized Euclidean distance as an evaluation criterion, exhibited potential deviations that could tend to misclassify samples with indistinct spectral features, and the confused points used for modeling might negatively impact prediction accuracy. Regarding the application of the FCM method to Sentinel-2 images, the “hard” classification results in the figures presented reasonable and expected distribution patterns, with each pixel classified into the dominant OWT. Although some pixels exhibited higher membership grades in a specific OWT, they remained small compared to those of other OWTs. As a result, the classification outcomes corresponded to the OWT with the maximum membership grade across all classes.

### 4.2. Performance of Blending CNN-RF Model

The predictions showed that the hybrid CNN-RF models, used as the base regressor, achieved better turbidity estimation performance. The CNN model’s internal filters continuously captured abundant local spatial and spectral features of the input data within the sliding window through the layer-by-layer convolutional structure. The deeper layers of the CNN integrated these local features to more effectively model and explicate the relationship between water turbidity and spectral reflectance. The sliding window size, an important parameter in CNN-based feature extraction, directly influences feature presentation, which, in turn, impacts model performance. This study determined 9 × 9 as the optimal window size for the input image patches, which can balance model accuracy and computational efficiency. The inversion maps showed that the adopted window size can reflect the fine spatial distribution details of turbidity. The RF method can effectively handle the high-dimensional data generated by the CNN model, which is more effective and useful for nonlinear regression estimation than the CNN’s internal linear activation functions. The hybrid CNN-RF model effectively integrated CNN’s feature extraction capability and RF’s data handling and regression prediction abilities, thereby serving as a base regressor for precise turbidity estimation. The proposed weighted blending CNN-RF model based on OWTs demonstrated outstanding advantages in terms of model performance and remote sensing image-based applications compared with the model without water classification and the unweighted model with OWTs. The proposed model also showed strong robustness and generalization ability. Furthermore, compared to the prediction accuracy achieved by machine learning and deep learning models for water turbidity in previous studies, the proposed blending CNN-RF model demonstrated superior performance (Table 4). The machine learning algorithms were more commonly applied. Li et al. (2023a) [14] and Li et al. (2023b) [64] achieved turbidity estimation across a large geographical spatial scale, which encompassed the entire national scope, while other studies focused primarily on specific water bodies.

Compared with unweighted models based on OWTs, the turbidity maps derived from the weighted blending model better maintained the continuous spatial distribution of turbidity. Specifically, each OWT-based CNN-RF model’s learnable parameters were updated to correspond to the specific OWT for turbidity estimation. As a result, the turbidity predictions were closer within specific water types but distinct across different OWTs. Consistently, the spatial distribution maps demonstrated that the differences in distribution patterns were also not significant among specific water types. Notably, blending the predictions of models for various OWTs through membership grade weights effectively and significantly reduced the uncertainty and deviation in the turbidity predictions by avoiding the rigid classification of water bodies into distinct types. The turbidity maps revealed that the typical water bodies with high turbidity were around croplands and human settlements. Human activities frequently cause soil disturbance, and domestic or agricultural wastewater might flow into these water bodies. These factors resulted in increased particulate matter concentrations or mesotrophic or eutrophic water, which, in turn, increased water turbidity.

However, the proposed blending turbidity estimation model had limitations related to its predictive accuracy, generalization capability, and application scope. Specifically, in this study, considering the abundance and quality of the dataset, we selected the appropriate Sentinel-2 images corresponding to the in situ data within a ±7-day period. Although the water quality of lakes and reservoirs in Northeast China is relatively stable, the time difference in image matching might lead to prediction inaccuracies. This is because it creates a mismatch between the turbidity measurements and the actual reflectance values at the time of image capture. Moreover, the sample points used for model construction were mainly collected from the water bodies in Northeast China. Despite capturing the typical reflectance characteristics of inland waters with varying turbidity, these samples were limited in the ability to adequately characterize the specific optical properties of waters in other geographic regions. This limitation, in turn, could affect the generalization capability and application scope of the proposed model. Furthermore, the proposed deep learning model was developed after optical water classification. This process could lead to a significant reduction in the number of samples available for each water type compared to the total dataset. Consequently, it was necessary to consider the risk of overfitting during model development, though it was not observed in this study. Thus, additional efforts can be devoted to the collection of in situ samples across several geographic regions with varying environmental conditions in subsequent studies. The expanded sample size and diversity were better able to support the construction of OWT-based deep learning models, which had the potential to enhance the performance and practical applicability of the developed model.

## 5. Conclusions

This study combined the spectral clustering of optical water types with the CNN-RF deep learning model for turbidity estimation using Sentinel-2 images. The acceptability and feasibility of the FCM method were verified to classify three optical water types based on spectral reflectance. The hybrid CNN-RF model successfully combined CNN’s proficiency in feature extraction with RF’s strengths in high-dimensional data processing and regression prediction, enabling it to serve as a base regressor for accurate turbidity estimation. A weighted sum of the turbidity predictions generated by the OWT-based CNN-RF model was computed, with the weight assigned to each OWT being the corresponding membership grade derived from the FCM clustering model. This approach effectively mitigates uncertainty and reduces prediction deviation by avoiding the rigid categorization of water bodies into discrete types. The proposed weighted blending CNN-RF model exhibited strong robustness and generalization ability, with the highest turbidity prediction accuracy (*R*^2^ = 0.900 and *RMSE* = 11.698 NTU). The turbidity maps for four typical water bodies derived from the proposed model better retained the continuous spatial distribution of turbidity and effectively revealed the water quality condition. The OWT-based weighted blending CNN-RF model may exhibit broad applicability to similar inland water bodies. Furthermore, it can be expanded to encompass data from additional sensors with spectral properties similar to the Sentinel-2 MSI sensor. These findings shed light on the application of deep learning models in water turbidity estimation and effectively enhance the remote sensing technology for water quality monitoring.

## Figures and Tables

**Table 1:** Statistical summary of turbidity for sampling points from 2018 to 2024. Mean, S.D., Min., and Max. are in NTU. *N* denotes the number of collected samples in the specific year.

Sampling Year	*N*	Mean (NTU)	S.D. (NTU)	Min. (NTU)	Max. (NTU)
2018	188	36.051	31.686	0.532	136.597
2019	51	15.604	13.859	0.372	73.780
2021	111	83.737	39.281	4.303	192.486
2022	231	38.443	36.574	0.159	187.197
2023	19	57.756	19.409	25.838	102.843
2024	68	20.475	10.698	7.821	67.077
Total	668	42.271	38.026	0.159	192.486

**Table 2:** Statistical summary of in situ water quality parameters for several OWTs. Min, Max, Mean, and STD denote the minimum, maximum, average, and standard deviation, respectively.

OWTs	Statistics	Turbidity (NTU)	Chla (μg/L)	TSM (mg/L)
1	Min	0.159	0.474	0.727
	Max	102.843	47.383	54.500
	STD	26.630	9.465	18.944
	Mean	27.975	8.807	25.271
2	Min	0.372	0.085	0.571
	Max	187.197	107.996	205.333
	STD	35.173	18.979	40.713
	Mean	42.010	12.360	33.735
3	Min	1.055	0.061	0.857
	Max	192.486	58.908	390.000
	STD	46.250	16.469	47.117
	Mean	57.214	15.590	50.607

**Table 3:** Calibrated performance of CNN-RF models based on different OWTs. *N* denotes the number of samples in the calibration dataset.

OWTs	*N*	*R* ^2^	*RMSE* (NTU)
1	120	0.955	5.632
2	226	0.942	8.419
3	121	0.953	10.180

**Table 4:** Comparison of quantitative turbidity estimation models in existing studies. *N* denotes the number of samples used for model development.

References		*N*	Turbidity Range (NTU)	Best Model	*R* ^2^	*RMSE* (NTU)
Ma et al. (2021)	[49]	187	0.83–112.26	GBDT	0.88	9.90
Wang et al. (2021)	[65]	94	21.30–140.80	ANN	0.87	10.83
Li et al. (2023a)	[14]	484	0.00–282.74	BP-TURB	0.88	4.42
Li et al. (2023b)	[64]	1081	0.15–262.57	RF	0.92	12.65
Leggesse et al. (2023)	[66]	286	0.27–344.00	RF	0.80	7.82
Yang et al. (2023)	[67]	263	60.00–100.00	GB	0.75	0.51
Zhang et al. (2024)	[68]	360	2.87–31.43	XGBoost	0.79	2.18
Singh et al. (2025)	[69]	220	3.22–576.00	RF	0.77	33.16
Kong et al. (2025)	[70]	168	1.20–8.10	RF	0.63	1.58
This study	-	668	0.16–192.49	CNN-RF	0.90	11.70

## Data Availability

The Google Earth Engine dataset is available at https://developers.google.com/earth-engine/datasets (accessed on 16 October 2024).
